# Association of tumors having Epstein–Barr virus in surrounding lymphocytes with poor prognosis

**DOI:** 10.1002/cam4.4967

**Published:** 2022-06-21

**Authors:** Norikazu Yogi, Genki Usui, Keisuke Matsusaka, Masaki Fukuyo, Ryoji Fujiki, Motoaki Seki, Shigetsugu Takano, Hiroyuki Abe, Teppei Morikawa, Tetsuo Ushiku, Masayuki Ohtsuka, Atsushi Kaneda

**Affiliations:** ^1^ Department of General Surgery, Graduate School of Medicine Chiba University Japan; ^2^ Department of Molecular Oncology, Graduate School of Medicine Chiba University Japan; ^3^ Department of Pathology, Graduate School of Medicine The University of Tokyo Tokyo Japan; ^4^ Department of Diagnostic Pathology NTT Medical Center Tokyo Tokyo Japan; ^5^ Department of Pathology Chiba University Hospital Chiba Japan; ^6^ Cancer Genomics Center Chiba University Hospital Chiba Japan

**Keywords:** Epstein–Barr virus (EBV), gastric cancer, lymphocyte, pancreatic cancer

## Abstract

Infection with certain viruses is an important cause of cancer. The Pan‐Cancer Analysis of Whole Genomes (PCAWG) Consortium recently analyzed the whole‐genome sequencing (WGS) data from 2656 cases across 21 cancer types, and indicated that Epstein–Barr virus (EBV) is detected in many different cancer cases at a higher frequency than previously reported. However, whether EBV‐positive cancer cases detected by WGS‐based screening correspond to those detected by conventional histopathological techniques is still unclear. In this study, to elucidate the involvement of EBV in various cancers, we reanalyzed the WGS data of the PCAWG cohort combined with the analysis of clinical samples of gastric and pancreatic cancer in our cohort. Based on EBV copy number in each case, we classified tumors into three subgroups: EBV‐High, EBV‐Low, and EBV‐Negative. The EBV‐High subgroup was found to be EBV‐positive in the cancer cells themselves, whereas the EBV‐Low subgroup was EBV‐positive in the surrounding lymphocytes. Further, the EBV‐Low subgroup showed a significantly worse prognosis for both gastric cancer and across cancer types. In summary, we classified tumors based on EBV copy number and found a unique cancer subgroup, EBV‐positive in the surrounding lymphocytes, which was associated with a poor prognosis.

## Graphical abstract

Considering the strong relationship between viruses and cancer, and the potential role of Epstein‐Barr virus (EBV) in different cancers, this study examined the involvement of EBV in various cancers based on the reanalysis whole‐genome sequencing data from the PCAWG cohort combined with the analysis of clinical samples of gastric and pancreatic cancer in our cohort. Based on EBV copy numbers in each case, we identified three subgroups of cancer: EBV‐High with EBV in the cancer cells, EBV‐Low with EBV in the surrounding lymphocytes, and EBV‐Negative subgroups. The EBV‐Low is a unique cancer subgroup showing a significantly worse prognosis in gastric cancer and across cancer types.

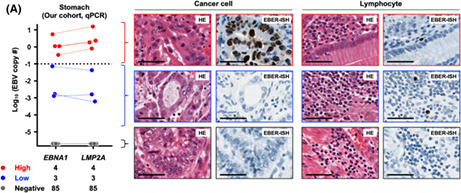

## 1 INTRODUCTION

Cancer arises through the accumulation of genetic and epigenetic aberrations caused by various environmental factors including viral and bacterial infection.^1^, ^2^, ^3^ The International Agency for Research on Cancer reported that among 14 million new patients with cancer per year, 2.2 million (15.4%) experience oncogenic infection.^4^ Viral tumorigenesis is a complex process, and cancer develops only in a small proportion of infected patients over a long period after the initial infection through multistep carcinogenesis.^5^ Despite improved public health and established preventive measures such as vaccines, understanding and controlling virus‐induced tumorigenesis remains an important challenge.

Epstein–Barr virus (EBV), or human gammaherpesvirus 4, is a widespread pathogen that causes infectious mononucleosis.^6^ Initial EBV infection occurs in early childhood through direct contact with saliva; once infected, EBV persists in host B cells throughout life.^7^ Although more than 90% of adults are EBV carriers through latent infection, EBV genomes, including latent genes such as *EBNA1* and *LMP2A*, cannot be confirmed in most human normal tissues because EBV persists only in a small proportion of B cells in pharynx and peripheral blood.^8^, ^9^

Infection‐induced carcinogenesis is an important aspect of EBV. In immunocompromised hosts, EBV causes opportunistic lymphoma, such as post‐transplant lymphoproliferative disorder.^10^, ^11^ In immunocompetent hosts, EBV induces other types of malignancies, such as Burkitt lymphoma,^12^ Hodgkin lymphoma,^13^ nasopharyngeal cancer,^14^ and gastric cancer (GC).^15^, ^16^ In EBV‐associated GC, epigenetic alterations play an essential role in tumorigenesis.^17^, ^18^ These epigenetic changes are specifically identified in cancer cells, which are positive for EBV, although virus causing epigenetic aberrations and initiating/promoting tumorigenesis might sometimes be lost entirely (“hit and run” hypothesis).^19^, ^20^ Surrounding normal epithelial cells in the mucosa, however, are not involved in EBV infection or such tumorigenic alteration of epigenome.^21^, ^22^ However, EBV involvement and its role in other cancers are yet to be fully elucidated.

The development of virus detection pipelines from human next‐generation sequencing data has provided new insights into viral associations with various cancers.^23^, ^24^, ^25^ Recently, Zapatka et al. analyzed the data of the Pan‐Cancer Analysis of Whole Genomes (PCAWG) cohort, including whole‐genome sequencing (WGS) data from 2656 cases across 21 cancer types, which was generated by the International Cancer Genome Consortium (ICGC) and The Cancer Genome Atlas (TCGA), and elucidated the association of viruses in various cancer types.^26^ Their report suggested EBV involvement in 18 cancer types, including 15 cancer types that are not reported to be associated with EBV. For example, EBV was detected in 3% of pancreatic cancer (PC) cases in the PCAWG cohort, whereas EBV‐associated PC is rarely encountered, with only four cases reported.^27^, ^28^, ^29^, ^30^ They also identified EBV in 35% of GC cases, which was much higher than the reported frequency of EBV‐associated GC at 7–15%.^31^, ^32^ Thus, whether EBV‐positive cancer cases detected by WGS‐based screening correspond to EBV‐associated cancers detected by conventional histopathological techniques remains controversial.

Here, to clarify the involvement of EBV in various cancer types, we reanalyzed the WGS data from the PCAWG cohort combined with an analysis of clinical samples of GC and PC in our cohort. Based on EBV copy number, we classified tumors into three subgroups and identified a unique cancer subgroup, EBV‐positive in the surrounding lymphocytes, which was associated with a poor prognosis.

## 2 MATERIALS AND METHODS

### 2.1 Clinical samples

Clinical samples of GC were collected from 92 patients who underwent surgical or endoscopic resection at the University of Tokyo Hospital or NTT Medical Center Tokyo. Clinical samples of PC were obtained from 105 patients who underwent surgical resection at Chiba University Hospital. The study design was approved by the ethics committees of the three hospitals, and written informed consent was obtained from each patient.

### 2.2 DNA extraction

DNA was extracted from formalin‐fixed paraffin‐embedded (FFPE) tissue samples of GC and PC using the QIAamp DNA FFPE Tissue Kit (Qiagen). All specimens were microscopically reviewed by two independent pathologists and macrodissected to enrich the tumor cell contents to greater than 50%.

### 2.3 PCAWG cohort data and calculation of EBV copy number

We reanalyzed 2656 cases across 21 cancer types from the PCAWG cohort, including 2727 cancer samples and 2627 normal samples. Transcriptome data, reverse phase protein array data, DNA methylation data, clinicopathological data, and survival data were obtained from the ICGC data portal (https://dcc.icgc.org) and the genomic data commons data portal (https://portal.gdc.cancer.gov). The number of reads mapped to EBV genome and human genome from WGS data was obtained from the previous report by Zapatka et al.^26^ The EBV copy number was calculated using the number of reads mapped to EBV genome, adjusted by the EBV genome size (1.7 × 10^5^ bp), and normalized by the number of reads mapped to human genome:

$$\mathrm{EBV}\;\text{copy number}=\frac{\text{Reads mapped to}\;\mathrm{EBV}\;\text{genome}/1.7\times 10^{5}}{\text{Reads mapped to human genome}/3.0\times 10^{9}}$$

For detection of EBV transcripts from GC cases in the PCAWG cohort, non‐human reads from RNA sequencing data were aligned to EBV reference genome data (NC_007605.1) and read counts per million read were calculated by StringTie tool v2.1.4.

### 2.4 DNA methylation analysis

We examined the DNA methylation data of GC cases in the PCAWG cohort, produced using the Infinium HumanMethylation450 BeadChip (Illumina). To classify GC cases by DNA methylation status and identify EBV‐associated GC, we used methylation data from our previous report on DNA methylation epigenotypes: EBV‐epigenotype, high‐epigenotype, and low‐epigenotype.^17^ We selected 204 Infinium probes as methylation markers: 53 EBV‐markers methylated in the EBV‐epigenotype, 79 high‐markers methylated in the EBV‐ and high‐epigenotypes, and 72 common‐markers methylated in all epigenotypes. We then analyzed DNA methylation data from GC cases in the PCAWG cohort by clustering analysis using R packages “Heatplus” and “amap.”

### 2.5 Gene set enrichment analysis (GSEA)

We analyzed the transcriptome data of GC and surrounding normal tissues in the PCAWG cohort using GSEA v4.1.0 software (Broad Institute). We compared the elevated or reduced molecular signatures in HALLMARK gene sets between EBV subgroups, and created a heatmap using the absolute log‐transformed *p*‐values with plus or minus signs indicating whether the signatures were elevated or reduced. The heatmap was drawn using R packages “Heatplus” and “amap.”

### 2.6 Quantitative PCR (qPCR) and calculation of EBV copy number

We performed qPCR using SYBR Green and CFX96 Touch Real‐Time PCR (Bio‐Rad Laboratories, Hercules, CA) to quantify EBV genomes (*EBNA1*, *LMP2A*) and a control region of the host genome (CTRL) in GC and PC clinical samples in our cohort. The EBV copy number was calculated from the *EBNA1* and *LMP2A* copy numbers, which were normalized by the CTRL copy number. CTRL is a unique region located on human chromosome 12 (from 6,538,560 to 6,538,637, hg38). The primer design information is shown in Table S1.

### 2.7 TA cloning and Sanger sequencing

PCR products for *EBNA1* and *LMP2A* were purified using the FastGene Gel/PCR Extraction Kit (NIPPON Genetics), and cloned into the pGEM‐T Easy Vector (Promega) by incubating at 16°C for 30 min using a DNA Ligation Kit (Takara Bio). XL1‐Blue Competent Cells (Agilent Technologies) were transformed with recombinant plasmids on LB‐ampicillin plates. The recombinant plasmids were then purified using the FastGene Plasmid Mini Kit (NIPPON Genetics), followed by colony PCR with SP6 and T7 promoter primers (Table S2). The amplified fragments were directly sequenced using a 3130 Genetic Analyzer (Thermo Fisher Scientific) with SP6 or T7 promoter primers.

### 2.8 In situ hybridization targeting EBER (EBER‐ISH)

We performed EBER‐ISH in FFPE tissues of GC and PC cases in our cohort using the VENTANA BenchMark ULTRA automated staining system (Roche) and an EBER probe (800–2842, Roche), as previously reported.^33^ We evaluated the stained slides using the Aperio ImageScope (Leica Biosystems Nussloch GmbH), and counted EBER‐ISH‐positive cells in 50 randomly selected 100‐μm^2^ fields.

### 2.9 Statistical analysis

The correlation between EBV‐Low samples in cancer tissues and in surrounding normal tissues was confirmed by Fisher's exact test. For EBV transcripts, *PD‐L1* gene expression, and PD‐L1 protein expression, we analyzed their association with EBV subgroups using Student's *t*‐test. The correlation between *EBNA1* and *LMP2A* copy number and EBER‐ISH‐positive cell counts was calculated using Spearman's rank correlation coefficient. The association between patient characteristics and EBV subgroups was analyzed using the χ^2^‐test, Fisher's exact test, Student's *t*‐test, ANOVA, Mann–Whitney U‐test, and Kruskal–Wallis test. Overall survival was estimated using the Kaplan–Meier method and compared using the log‐rank test. The hazard ratio (HR) and 95% confidence interval (CI) were computed using a Cox proportional hazard model adjusted for age, sex, EBV copy number, and cancer type. All statistical analyses were performed using JMP Pro 15.1.0 (SAS Institute). Statistical significance was set at *p* < 0.05.

## 3 RESULTS

### 3.1 Classification of GC and PC cases in the PCAWG cohort based on EBV copy number

**FIGURE 1:**
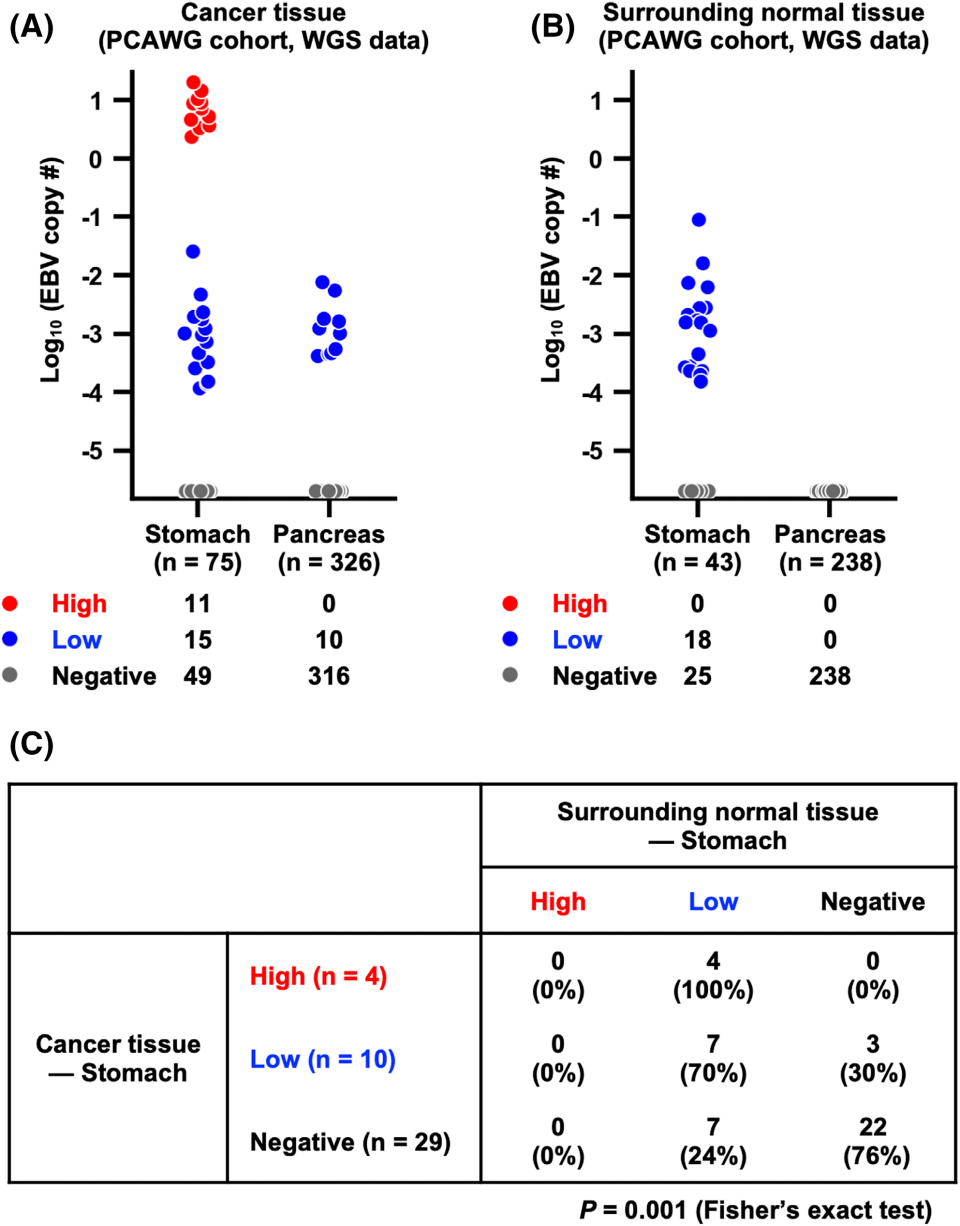
EBV copy number analysis for gastric cancer (GC) and pancreatic cancer (PC) in the PCAWG cohort. (A) Distribution of EBV copy numbers in GC tissues (*n* = 75) and PC tissues (*n* = 326) using whole‐genome sequencing (WGS) data of the PCAWG cohort. *Red dots*, samples with high copy numbers of EBV. *Blue dots*, samples with low copy numbers of EBV. *Gray dots*, samples without EBV. (B) Distribution of EBV copy numbers in the surrounding normal tissues of GC (*n* = 43) and PC (*n* = 238) using WGS data of the PCAWG cohort. *Blue dots*, samples with low copy numbers of EBV. *Gray dots*, samples without EBV. (C) Correlation of EBV copy number between cancer tissues and surrounding normal tissues in GC cases from the PCAWG cohort (*n* = 43). Both of EBV‐High and EBV‐Low cases in cancer tissues showed low copy numbers of EBV in surrounding normal tissues (*p* = 0.001).

To elucidate the involvement of EBV infection in various cancers, we reanalyzed the WGS data from the PCAWG cohort reported by Zapatka et al.^26^ We first focused on GC and PC cases and calculated the EBV copy number. Among the 75 GC samples, 49 contained no EBV genomes, whereas 11 showed markedly high copy numbers of EBV at more than 2.3 × 10^0^ and 15 showed relatively low copy numbers ranging from 1.2 × 10^−4^ to 2.5 × 10^−2^ (Figure 1A). Among the 326 PC samples, 316 contained no EBV genomes, whereas 10 showed low copy numbers of EBV ranging from 4.1 × 10^−4^ to 7.6 × 10^−3^ (Figure 1A). We also calculated the EBV copy number in the surrounding normal tissues of GC and PC. Among the 43 samples of GC surrounding normal tissues, 25 showed no EBV genomes, whereas 18 showed low copy numbers of EBV ranging from 1.5 × 10^−4^ to 8.9 × 10^−3^ (Figure 1B). None of the PC surrounding normal tissues contained EBV genomes (Figure 1B).

Therefore, we classified the samples into three subgroups based on their EBV copy number: EBV‐High, EBV‐Low, and EBV‐Negative. Samples with EBV copy numbers more than 10^−1^ were classified as EBV‐High, samples with EBV copy numbers less than 10^−1^ were classified as EBV‐Low, and samples without EBV genomes were classified as EBV‐Negative. We applied this classification based on EBV copy number to the GC and PC cases in the PCAWG cohort. The GC samples included 11 EBV‐High, 15 EBV‐Low, and 49 EBV‐Negative samples, and the PC samples included 10 EBV‐Low and 316 EBV‐Negative samples (Figure 1A). Among the GC surrounding normal tissues, 18 EBV‐Low and 25 EBV‐Negative samples were detected; however, no EBV‐High samples were found (Figure 1B). Only EBV‐Negative samples were detected among the PC surrounding normal tissues (*n* = 238) (Figure 1B).

Using GC samples and their paired normal samples, we investigated the association of EBV copy number in cancer tissues with that in surrounding normal tissues. Most EBV‐Low cancer samples showed low copy numbers of EBV in their surrounding normal tissues (Figure 1C), suggesting that low copy numbers of EBV are derived from the surrounding normal tissues. Interestingly, EBV‐High cancer samples also showed low copy numbers of EBV in their surrounding normal tissues (Figure 1C).

We next analyzed RNA sequencing data of 30 GC cases from the PCAWG cohort, including seven EBV‐High, four EBV‐Low, and 19 EBV‐Negative cases. Significantly higher levels of EBV transcripts were observed in EBV‐High cases than those in EBV‐Low and EBV‐Negative cases (*p* < 1 × 10^−4^) (Figure S1A, B). Although EBV transcript levels in EBV‐Low cases were low, they were still significantly higher than those in EBV‐Negative cases (*p* = 4 × 10^−4^) (Figure S1A, B). EBV genes corresponding to latency type I, such as BART, EBER, LMP2A, and EBNA1, were highly expressed in EBV‐High cases (Figure S1C).

### 3.2 Classification of various cancer and normal tissues in the PCAWG cohort based on EBV copy number

We then classified various cancer tissues and their paired normal tissues in the PCAWG cohort into the three EBV subgroups. Among the 2727 cancer samples, EBV‐High samples were identified only in GC tissues (*n* = 11) and in lymphoma tissue (*n* = 1) (Figure S2A), which are well‐known EBV‐associated cancers. We found 130 EBV‐Low samples in various cancer types, including those not reported as EBV‐associated cancers. These findings suggest that EBV‐High samples corresponded to EBV‐associated cancers, and that EBV‐Low samples were a unique subgroup distinct from EBV‐associated cancers.

In the analysis of paired normal tissues (*n* = 2627), 68 EBV‐Low samples were found in various normal tissues, whereas EBV‐High samples were detected only in the paired normal tissues of myeloid leukemia (*n* = 1) and ovarian cancer (*n* = 16) (Figure S2B). Among the paired normal tissues of myeloid leukemia, the EBV‐High sample was derived from saliva, which sometimes contains high copy numbers of EBV^34^. Among the paired normal tissues of ovarian cancer, EBV‐High samples were derived from normal blood controls immortalized by EBV. Thus, EBV‐High samples were not observed in paired normal tissues, except for some specific cases of myeloid leukemia and ovarian cancer.

### 3.3 Association between EBV subgroups and GC molecular characteristics

**FIGURE 2:**
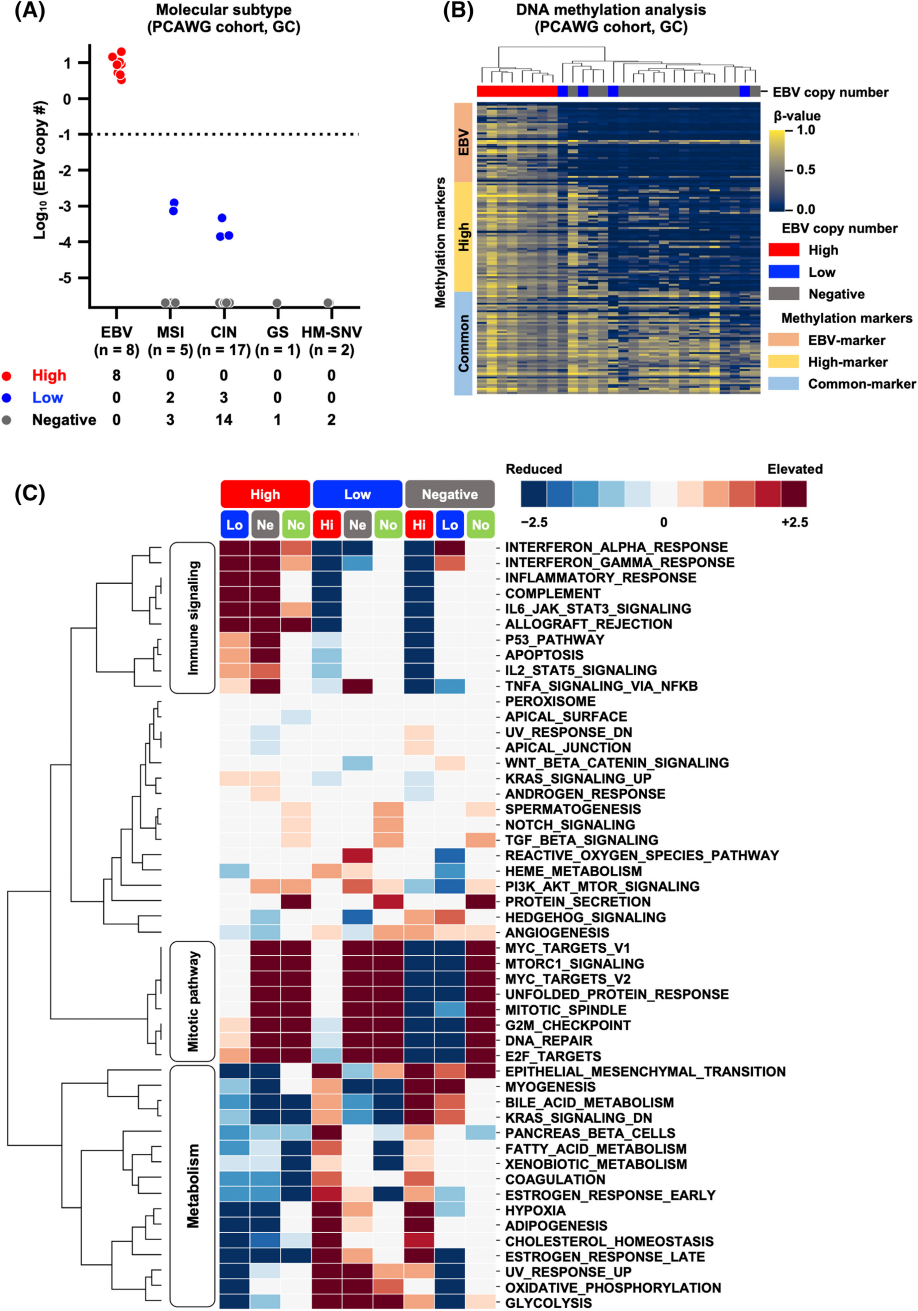
Association between EBV subgroups and gastric cancer (GC) molecular characteristics in the PCAWG cohort. (A) Association between EBV copy number and GC molecular subtypes: EBV, MSI, CIN, GS, and HM‐SNV. All EBV‐High samples (*n* = 8) belonged to the EBV subtype. The horizontal dotted line shows the cut‐off value. (B) Association between EBV copy number and DNA methylation epigenotypes. Clustering analysis was performed using 204 Infinium probes, including 53 EBV‐markers, 79 high‐markers, and 72 common‐markers. All EBV‐High samples (*n* = 8) belonged to the EBV‐epigenotype. *Top*, EBV copy number. *Left*, methylation markers. (C) Association between EBV subgroups and enriched gene sets. Gene set enrichment analysis was performed with a comparison of all combinations of EBV‐High, EBV‐Low, EBV‐Negative, and surrounding normal tissues. Each column indicates which gene sets were elevated (*red*) or reduced (*blue*) when comparing the two subsets shown at the top of the heatmap. For example, the first column shows a comparison of enriched gene sets between the EBV‐High and EBV‐Low subgroups. *Hi*, EBV‐High; *Lo*, EBV‐Low; *Ne*, EBV‐Negative; *No*, surrounding normal tissues.

To explore the characteristics of EBV‐High and EBV‐Low samples more closely, we used GC samples from the PCAWG cohort for further analysis. The PCAWG cohort included 33 GC samples from the TCGA cohort (TCGA‐STAD); we thus used the annotation data of five molecular subtypes in TCGA‐STAD: EBV, MSI, CIN, GS, and HM‐SNV^35^, which confirmed the relationship between EBV copy number and GC molecular subtypes (Figure 2A). As expected, all eight EBV‐High samples belonged to the EBV subtype. Among the five EBV‐Low samples, two were included in the MSI subtype, and three were included in the CIN subtype.

We also conducted a clustering analysis for DNA methylation data using methylation markers reported by Matsusaka et al.^17^ (Figure 2B). All eight EBV‐High samples were found to be involved in the EBV‐epigenotype with extremely high methylation, including the EBV‐markers. The two EBV‐Low samples with MSI subtype belonged to the high‐epigenotype, and the two EBV‐Low samples with CIN subtype belonged to the low‐epigenotype.

To determine the underlying differences between the three subgroups based on EBV copy number, we performed GSEA using the HALLMARK gene sets (Figure 2C). Hierarchical clustering of the enriched gene sets revealed several specific patterns, including elevation of mitotic pathways, such as *MYC* targets, mitotic spindle, and DNA repair, in all cancer tissues compared with those in normal tissues. This result was consistent with a previous report from the TCGA Research Network.^36^ The unique gene sets elevated in the EBV‐High samples were related to immune cell signaling, including interferon (IFN) response, inflammatory response, and IL‐6/STAT3 signaling. *PD‐L1*, a member of HALLMARK IFN‐γ response gene sets, were highly expressed in the EBV‐High samples in both transcriptome level (Figure S3A) and protein level (Figure S3B) compared with that in the EBV‐Low and EBV‐Negative samples. These gene sets and *PD‐L1* gene expression were also reported to be enriched in the EBV subtype compared with those in other GC subtypes.^36^ EBV‐High samples exhibited decreased expression of metabolism‐associated pathways, including bile acid metabolism, adipogenesis, and cholesterol homeostasis. Genes associated with IFN‐α and IFN‐γ responses were decreased in EBV‐Low samples compared with those in EBV‐High and EBV‐Negative samples.

### 3.4 Classification of GC and PC cases in our cohort based on EBV copy number and EBER‐ISH

**FIGURE 3:**
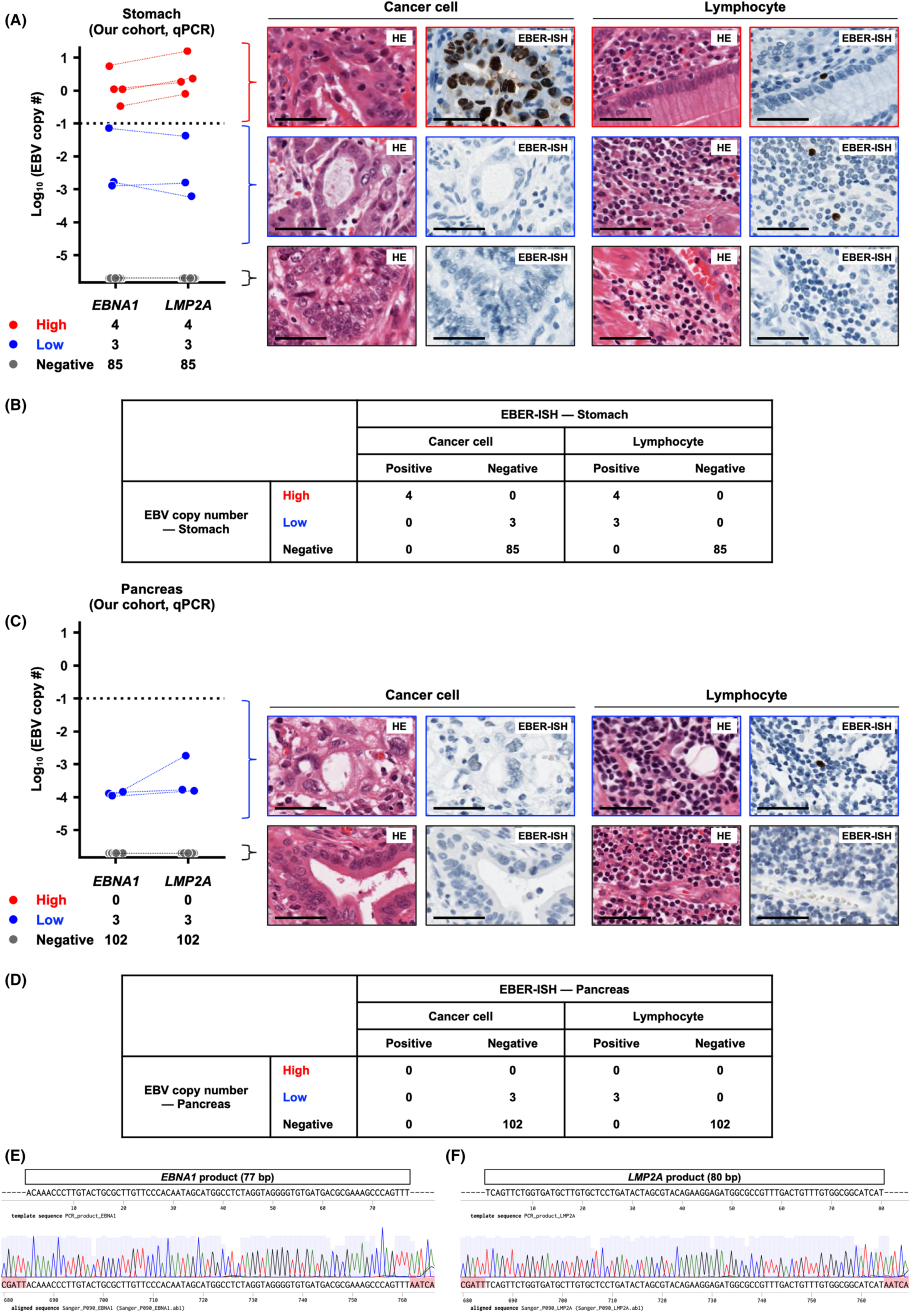
EBV copy number analysis for gastric cancer (GC) and pancreatic cancer (PC) in our cohort. (A) EBV copy number analysis based on quantitative PCR (qPCR) and in situ hybridization targeting EBER (EBER‐ISH) in GC (*n* = 92). The left panel shows the distribution of EBV copy numbers with the cut‐off value indicated by the horizontal dotted line. *Red dots*, samples with high copy numbers of EBV. *Blue dots*, samples with low copy numbers of EBV. *Gray dots*, samples without EBV. The right panel shows the representative hematoxylin and eosin staining (HE) and EBER‐ISH paired images of EBV‐High, EBV‐Low, and EBV‐Negative samples in cancer cells and surrounding lymphocytes, respectively, top to bottom. *Scale bars*, 50 μm. (B) Association between EBV copy number and EBER‐ISH in GC. EBV‐High samples were positive for EBER‐ISH in both cancer cells and surrounding lymphocytes, whereas EBV‐Low samples were positive for EBER‐ISH in only surrounding lymphocytes. (C) EBV copy number analysis based on qPCR and EBER‐ISH in PC (*n* = 105). The left panel shows the distribution of EBV copy numbers with the cut‐off value indicated by the horizontal dotted line. *Blue dots*, samples with low copy numbers of EBV. *Gray dots*, samples without EBV. The right panel shows the representative HE and EBER‐ISH paired images of EBV‐Low and EBV‐Negative samples in cancer cells and surrounding lymphocytes, respectively, top to bottom. *Scale bars*, 50 μm. (D) Association between EBV copy number and EBER‐ISH in PC. EBV‐Low samples were positive for EBER‐ISH in only surrounding lymphocytes. (E) Direct sequencing of the *EBNA1* product. (F) Direct sequencing of the *LMP2A* product.

To validate the classification based on EBV copy number in our cohort, we performed qPCR for EBV genomes (*EBNA1*, *LMP2A*) and calculated the EBV copy number in 92 GC and 105 PC clinical samples. Simultaneously, we performed EBER‐ISH and confirmed the distribution of EBV in each specimen. Among the 92 GC clinical samples, four belonged to the EBV‐High subgroup with high copy numbers of EBV at more than 3.4 × 10^−1^, three belonged to the EBV‐Low subgroup with low copy numbers ranging from 6.1 × 10^−4^ to 4.2 × 10^−2^, and 85 belonged to the EBV‐Negative subgroup (Figure 3A, B). In all four EBV‐High samples, EBER‐ISH revealed that cancer cells were positive for EBV (Figure 3A, B), confirming EBV‐High samples as EBV‐associated GC. Furthermore, in EBV‐High samples, surrounding lymphocytes were also positive for EBV (Figure 3A, B), consistent with the results of the analysis for the PCAWG cohort showing that EBV‐High cancer samples contained low copy numbers of EBV in their surrounding normal tissues (Figure 1C). In all three EBV‐Low samples, cancer cells were negative for EBV, and the unique origin of EBV was surrounding lymphocytes (Figure 3A, B). This finding also matched the results of the analysis for the PCAWG cohort showing that the low copy numbers of EBV observed in the EBV‐Low cancer samples were derived from the surrounding normal tissues. (Figure 1C).

Among the 105 PC clinical samples, three belonged to the EBV‐Low subgroup with low copy numbers of EBV ranging from 1.6 × 10^−4^ to 1.8 × 10^−3^, and 102 belonged to the EBV‐Negative subgroup; however, no samples belonged to the EBV‐High subgroup (Figure 3C, D). In all three EBV‐Low samples, cancer cells were negative for EBV, and surrounding lymphocytes were positive for EBV (Figure 3C, D).

We then performed counts of EBER‐ISH‐positive cells in GC (Figure S4A) and PC (Figure S4B) clinical samples. *EBNA1* and *LMP2A* copy numbers were positively correlated with EBER‐ISH‐positive cell counts (*EBNA1* copy number: ρ = 0.90, *p* = 4 × 10^−4^; *LMP2A* copy number: ρ = 0.95, *p* < 1 × 10^−4^) (Figure S4C, D).

As the *EBNA1* and *LMP2A* copy numbers detected in the EBV‐Low samples were quite small, we performed Sanger sequencing of the PCR products from all EBV‐Low samples and confirmed that the sequences of the PCR products were consistent with those of *EBNA1* and *LMP2A* (Figure 3E, F).

### 3.5 Association between EBV subgroups, clinicopathological characteristics, and prognosis

The clinicopathological characteristics of EBV‐High, EBV‐Low, and EBV‐Negative cases in the PCAWG cohort and our cohort are shown for GC cases (Table 1) and PC cases (Table 2). In both GC and PC cases, there were no significant differences in age, sex, histological type, primary tumor status, regional lymph node status, and pathological stage between the EBV subgroups, except for EBV copy number.

**TABLE 1:** Clinicopathological characteristics in the PCAWG cohort and our cohort—stomach

	PCAWG cohort				Our cohort			
	EBV‐high (*n* = 11)	EBV‐low (*n* = 15)	EBV‐negative (*n* = 48)	*p*‐value	EBV‐high (*n* = 4)	EBV‐low (*n* = 3)	EBV‐negative (*n* = 85)	*p*‐value
Age								
Years (mean ± SD)	60.0 ± 12.7	63.5 ± 12.2	66.1 ± 12.0	0.3	66.0 ± 15.1	64.6 ± 9.9	66.9 ± 9.1	0.9
Sex								
Male	11 (100%)	10 (67%)	35 (73%)	0.1	4 (100%)	3 (100%)	72 (85%)	1
Female	0	5 (33%)	13 (27%)		0	0	11 (13%)	
Unknown	0	0	0		0	0	2 (2%)	
Histological type								
Papillary adenocarcinoma	1 (9%)	2 (13%)	0	0.2	0	0	1 (1%)	0.3
Tubular adenocarcinoma	2 (18%)	4 (27%)	14 (29%)		2 (50%)	3 (100%)	67 (79%)	
Mucinous adenocarcinoma	0	0	3 (6%)		0	0	0	
Poorly cohesive carcinoma	2 (18%)	4 (27%)	14 (29%)		2 (50%)	0	16 (19%)	
Unknown	6 (55%)	5 (33%)	17 (35%)		0	0	1 (1%)	
Primary tumor status								
T1	2 (18%)	1 (7%)	3 (6%)	0.3	3 (75%)	2 (67%)	65 (76%)	0.4
T2	0	3 (20%)	13 (27%)		0	1 (33%)	4 (5%)	
T3	5 (45%)	8 (53%)	18 (38%)		1 (25%)	0	7 (8%)	
T4	4 (36%)	2 (13%)	14 (29%)		0	0	8 (9%)	
Unknown	0	1 (7%)	0		0	0	1 (1%)	
Regional lymph node status								
N0	3 (27%)	4 (27%)	13 (27%)	0.7	3 (75%)	2 (67%)	70 (82%)	0.3
N1	3 (27%)	7 (47%)	14 (29%)		0	0	6 (7%)	
N2	2 (18%)	1 (7%)	12 (25%)		0	1 (33%)	3 (4%)	
N3	3 (27%)	2 (13%)	9 (19%)		1 (25%)	0	5 (6%)	
Unknown	0	1 (7%)	0		0	0	1 (1%)	
Pathological stage								
I	1 (9%)	0	8 (17%)	0.4	3 (75%)	2 (67%)	69 (81%)	0.3
II	2 (18%)	5 (33%)	12 (25%)		0	1 (33%)	5 (6%)	
III	5 (45%)	3 (20%)	14 (29%)		1 (25%)	0	10 (12%)	
IV	0	2 (13%)	3 (6%)		0	0	0	
Unknown	3 (27%)	5 (33%)	11 (23%)		0	0	1 (1%)	
EBV copy number								
WGS (median)	7.2	9.5 × 10^−4^	0.0	< 1 × 10^−4^	NA	NA	NA	NA
qPCR, *EBNA1* (median)	NA	NA	NA	NA	1.1	1.7 × 10^−3^	0.0	< 1 × 10^−4^
qPCR, *LMP2A* (median)	NA	NA	NA	NA	2.1	1.6 × 10^−3^	0.0	< 1 × 10^−4^

For age, the association with EBV subgroups was analyzed by ANOVA. For sex, histological type, primary tumor status, regional lymph node status, and pathological stage, the association was analyzed by Fisher's exact test. For EBV copy number, the association was analyzed by Kruskal–Wallis test.

Abbreviations: NA, not available.

**TABLE 2:** Clinicopathological characteristics in the PCAWG cohort and our cohort—pancreas

	PCAWG cohort			Our cohort		
	EBV‐low (*n* = 10)	EBV‐negative (*n* = 231)	*p*‐value	EBV‐low (*n* = 3)	EBV‐negative (*n* = 102)	*p*‐value
Age						
Years (mean ± SD)	67.5 ± 10.1	65.5 ± 11.3	0.6	75.0 ± 4.0	69.7 ± 0.9	0.4
Sex						
Male	5 (50%)	114 (49%)	0.1	1 (33%)	62 (61%)	0.3
Female	5 (50%)	115 (50%)		2 (67%)	40 (39%)	
Unknown	0	2 (1%)		0	0	
Histological type						
Well‐differentiated carcinoma	0	24 (10%)	0.7	1 (33%)	30 (29%)	0.5
Moderately differentiated carcinoma	5 (50%)	115 (50%)		2 (67%)	32 (31%)	
Poorly differentiated carcinoma	5 (50%)	76 (33%)		0	37 (36%)	
Undifferentiated carcinoma	0	10 (4%)		0	0	
Adenosquamous carcinoma	0	1 (0.4%)		0	3 (3%)	
Unknown	0	5 (2%)		0	0	
Primary tumor status						
T1	0	3 (1%)	1	0	4 (4%)	1
T2	0	11 (5%)		0	1 (1%)	
T3	2 (20%)	74 (32%)		3 (100%)	92 (90%)	
T4	0	2 (1%)		0	5 (5%)	
Unknown	8 (80%)	141 (61%)		0	0	
Regional lymph node status						
N0	1 (10%)	30 (13%)	0.6	0	19 (19%)	0.4
N1	1 (10%)	59 (26%)		3 (100%)	83 (81%)	
Unknown	8 (80%)	142 (61%)		0	0	
Pathological stage						
I	0	8 (3%)	1	0	3 (3%)	1
II	8 (80%)	121 (52%)		3 (100%)	90 (88%)	
III	0	4 (2%)		0	4 (4%)	
IV	0	3 (1%)		0	5 (5%)	
Unknown	2 (20%)	95 (41%)		0	0	
EBV copy number						
WGS (median)	1.1 × 10^−3^	0.0	< 1 × 10^−4^	NA	NA	NA
qPCR, *EBNA1* (median)	NA	NA	NA	1.3 × 10^−4^	0.0	< 1 × 10^−4^
qPCR, *LMP2A* (median)	NA	NA	NA	1.7 × 10^−4^	0.0	< 1 × 10^−4^

For age, the association with EBV subgroups was analyzed by Student's *t*‐test. For sex, histological type, primary tumor status, regional lymph node status, and pathological stage, the association was analyzed by χ^2^‐test and Fisher's exact test. For EBV copy number, the association was analyzed by Mann–Whitney U‐test.

Abbreviations: NA, not available.

**FIGURE 4:**
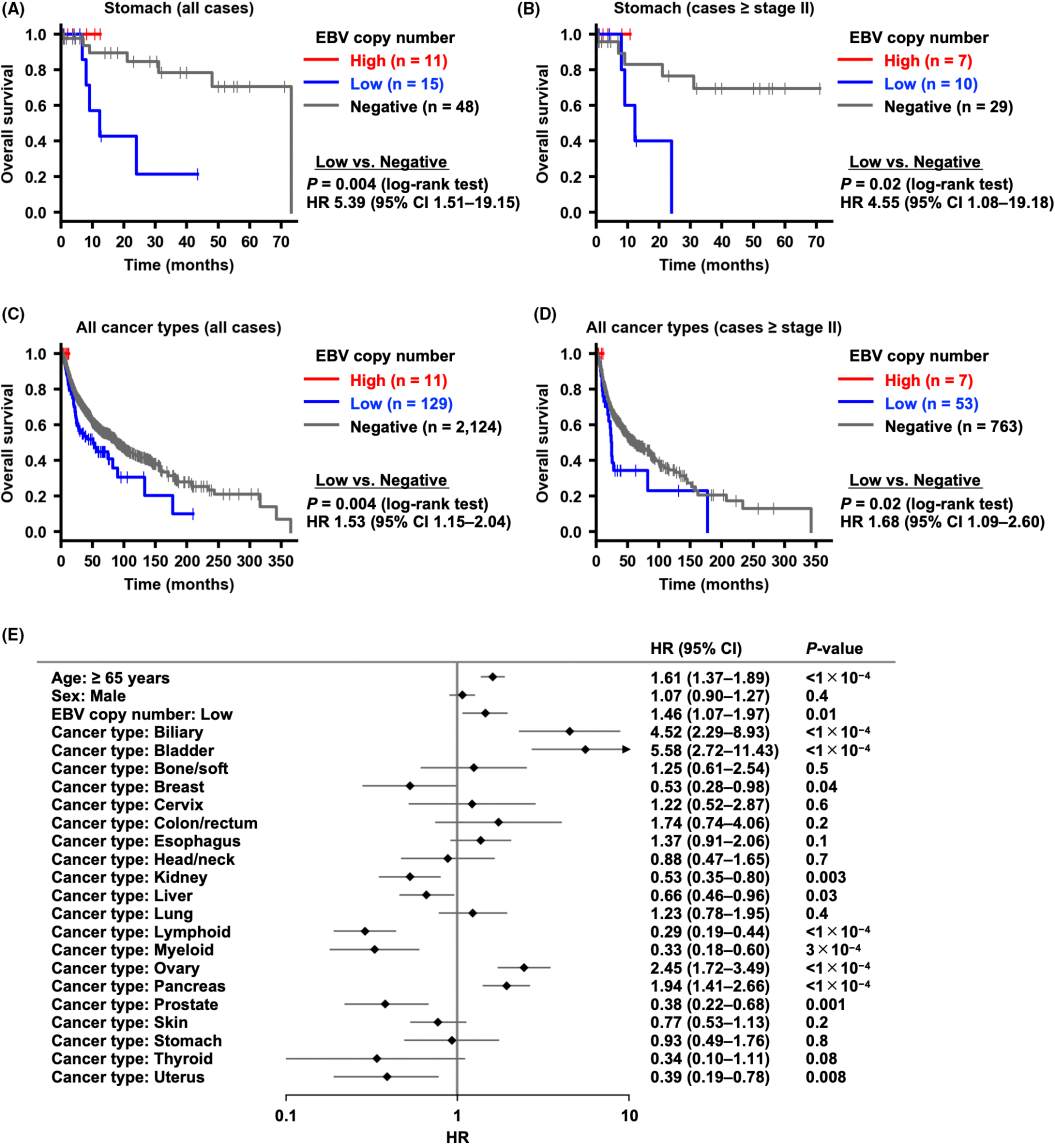
Association between EBV subgroups and prognosis in the PCAWG cohort. (A) Comparison of overall survival in gastric cancer (GC) (*n* = 74, all stages) between the EBV‐Low and EBV‐Negative subgroups. (B) Comparison of overall survival in GC (*n* = 46, stage II, III, and IV) between the EBV‐Low and EBV‐Negative subgroups. (C) Comparison of overall survival in all cancer types (*n* = 2264, all stages) between the EBV‐Low and EBV‐Negative subgroups. (D) Comparison of overall survival in all cancer types (*n* = 823, stage II, III, and IV) between the EBV‐Low and EBV‐Negative subgroups. (E) Multivariate analysis of overall survival in all cancer types (*n* = 2264) for age, sex, EBV copy number, and cancer type. The hazard ratio for cancer type was calculated by using CNS tumors as a reference. *Bone/soft*, bone and soft tissue; *CNS*, central nervous system; *HR*, hazard ratio; *CI*, confidence interval.

We then compared the overall survival of GC cases in the PCAWG cohort between the EBV subgroups and found that EBV‐Low cases showed a significantly worse prognosis than EBV‐Negative cases (*p* = 0.004) (Figure 4A). Even after excluding the cases with pathological stage I, the overall survival in EBV‐Low cases was still shorter than that in EBV‐Negative cases (*p* = 0.02) (Figure 4B).

We also compared the overall survival of 2264 cases across cancer types in the PCAWG cohort between the EBV subgroups. EBV‐Low cases showed a significantly worse prognosis than EBV‐Negative cases (*p* = 0.004) (Figure 4C). Even after excluding the cases with pathological stage I, the overall survival in EBV‐Low cases was still shorter than that in EBV‐Negative cases (*p* = 0.02) (Figure 4D). Multivariate analysis using the Cox proportional hazard model showed that the EBV‐Low cases were independently associated with a poor prognosis after adjusting for age, sex, and cancer type (HR 1.46, 95% CI 1.07–1.97, *p* = 0.01) (Figure 4E).

## 4 DISCUSSION

In this study, we performed an integrated analysis for the PCAWG cohort and our clinical cohort. Based on EBV copy number, EBV‐positive tumors were classified into EBV‐High and EBV‐Low subgroups. The EBV‐High subgroup corresponded to EBV‐associated cancers, whereas the EBV‐Low subgroup was observed in most cancer types and associated with EBV‐positive surrounding lymphocytes. The EBV‐Low subgroup showed a worse prognosis for both GC and across cancer types.

In the PCAWG cohort, EBV was reported to be detected in 35% of GC cases and in 3% of PC cases.^26^ However, we found that the distribution of EBV copy number was bimodal and that only EBV‐High cases corresponded to EBV‐associated cancers. The frequency of EBV‐High cases in GC was only 14.7%, concurring with the reported frequency of EBV‐associated GC at 15%.^32^ We observed no EBV‐High cases in PC, consistent with the fact that EBV‐associated PC is a rare tumor, with only four cases reported.^27^, ^28^, ^29^, ^30^ We also found one EBV‐High lymphoma sample derived from a patient with post‐transplant lymphoproliferative disorder, which is a well‐known complication after transplantation induced by EBV.^10^, ^11^ Further, EBV‐High GC cases exhibited the molecular characteristics of EBV‐associated GC, such as DNA hypermethylation in the promoter region^17^ and enrichment of immune cell signaling in transcriptome analysis.^36^ Finally, we identified that the cancer cells were EBER‐ISH‐positive in EBV‐High cases from our cohort. Thus, the EBV‐High cases were confirmed as EBV‐associated cancers, and the frequency of EBV involvement in various cancers was lower than that reported by Zapatka et al.^26^

EBV‐Low cases were observed across 18 cancer types from the PCAWG cohort, suggesting the presence of EBV‐positive lymphocytes in various cancers. EBV is known to latently infect a limited number of lymphocytes in the pharynx and peripheral blood^8^, ^9^; however, the significance of EBV in the tumor‐surrounding lymphocytes is still unclear. As an initial approach, it is necessary to properly detect cases with EBV‐positive lymphocytes. In this study, we classified tumors and isolated EBV‐Low cases by calculating the EBV copy number from WGS data. Selitsky et al. also analyzed the RNA sequencing data from TCGA and found a low level of EBV transcriptome in 13 tumor types, suggesting the association with EBV‐positive lymphocytes.^37^ Virus detection using next‐generation sequencing data is effective in identifying low copy numbers of EBV; further comprehensive EBV screening might allow a detailed analysis of the subgroup that is EBV‐positive in the surrounding lymphocytes.

Interestingly, EBV‐Low cases showed a significantly worse prognosis than EBV‐Negative cases for both GC and all cancer types. Ohashi et al. previously identified the presence of EBV in non‐neoplastic lymphocytes adjacent to diffuse large B‐cell lymphoma and found that EBV‐positive non‐neoplastic lymphocytes correlated with a poor prognosis.^38^ Although the mechanism contributing to the poor prognosis in EBV‐Low cases remains unclear, our findings suggested that the host immune status might be associated with unfavorable outcomes. In the present study, GSEA revealed that EBV‐Low cases showed significant decreases in IFN‐α and IFN‐γ responses compared with EBV‐High and EBV‐Negative cases. IFN‐α, a type I IFN, is mainly involved in innate immunity associated with antiviral and anti‐cancer responses.^39^, ^40^ IFN‐γ, the only member of the type II class of IFNs, is a critical cytokine for both innate and adaptive immunity, and promotes cytotoxic T lymphocyte‐induced anti‐tumor immunity.^40^ Reduced expression of these IFN‐related genes is associated with poor prognosis and tumor progression in breast cancer, cervical cancer, and melanoma.^41^, ^42^, ^43^ Furthermore, IFN‐γ is also essential for regulating EBV reactivation.^44^, ^45^, ^46^ Therefore, in EBV‐Low cases, immunosuppression induced by decreases in IFN‐α and IFN‐γ responses might lead to poor prognosis and infiltration of EBV‐positive lymphocytes. EBV‐positive lymphocytes were also observed in EBV‐High cases, and these EBV‐positive lymphocytes might be associated with EBV‐infection to epithelial cells in gastric mucosa transforming them to clonal growth as previously described.^16^ EBV‐associated GC is known to retain EBV‐specific cellular immunity,^47^ and immune cell signaling were activated in EBV‐High cases in the present study. Thus, in terms of immune status, EBV‐High and EBV‐Low cases are suggested to exhibit different characteristics.

A limitation of this study might be the sample size, especially the number of EBV‐Low GC and PC cases in our cohort. While we analyzed 92 cases of GC and 105 cases of PC, only three cases each were found to be EBV‐Low. Upon analyzing more than 1000 cases of GC and PC, we may better evaluate the prognostic impact of EBV in the surrounding lymphocytes specific for each cancer type. Further studies, together with functional analysis, could clarify the mechanism leading to the poor prognosis in the EBV‐Low subgroup.

In summary, we examined the EBV copy number in the PCAWG cohort as well as in our cohort, and classified tumors into three EBV subgroups. We further identified a unique cancer subgroup, EBV‐positive in the surrounding lymphocytes, which was associated with a significantly poor prognosis.

## AUTHORS’ CONTRIBUTIONS

N.Y., G.U., and A.K. carried out study conception and design. N.Y., G.U., K.M., M.F., M.S., and A.K. carried out method development. N.Y., G.U., M.F., R.F., S.T., H.A., T.M., T.U., and M.O. contributed to data acquisition. N.Y., G.U., K.M., M.F., M.S., and A.K. carried out data analysis and interpretation. N.Y., G.U., M.F., R.F., M.S., T.M., and A.K. were involved in writing and reviewing the manuscript. G.U. and A.K. carried out study supervision.

## Funding information

This work was supported by the Japan Agency for Medical Research and Development [grant number 21cm0106510h0006 to A.K.]; Japan Society for the Promotion of Science [grant number 19H03726 to A.K.]; Global and Prominent Research, Chiba University [grant number 2018‐Y9 to A.K.]; and Takeda Science Foundation [Specific Research Grant to A.K.].

## CONFLICT OF INTEREST DISCLOSURE

All authors have no conflict of interest.

## ETHICS APPROVAL AND PATIENT CONSENT STATEMENT

The study design was approved by the ethics committees of Chiba University Hospital, the University of Tokyo Hospital, and NTT Medical Center Tokyo. Written informed consent was obtained from each patient.

## Supporting information


**Supporting Information S1** Supplementary Table S1. Primers for quantitative PCR.
Supplementary Table S2. Primers for colony PCR and Sanger sequencing.
upplementary Figure S1. Association between EBV transcripts and EBV copy number in the PCAWG cohort.
Supplementary Figure S2. EBV copy number analysis for various cancer tissues and their paired normal tissues from the PCAWG cohort.
Supplementary Figure S3. *PD‐L1* gene expression and PD‐L1 protein expression in each EBV subtype.
Supplementary Figure S4. Association between in situ hybridization targeting EBER (EBER‐ISH)‐positive cell counts and EBV copy number in our cohort.

## Data Availability

Data availability statement Sequencing data from the PCAWG cohort are publicly available at the ICGC data portal (https://dcc.icgc.org) and the genomic data commons data portal (https://portal.gdc.cancer.gov). All other data supporting the findings of this study are available from the corresponding author upon reasonable request.
