# Supplementary material for: Association of tumors having Epstein–Barr virus in surrounding lymphocytes with poor prognosis
Source: Cancer Med. 2022 Jun 21;12(2):1122–36. doi: 10.1002/cam4.4967 (PMC9883551; doi:10.1002/cam4.4967)
Supplement: Supplementary file 1 — Supporting Information S1 Supplementary Table S1. Primers for quantitative PCR. Supplementary Table S2. Primers for colony PCR and Sanger sequencing. upplementary Figure S1. Association between EBV transcripts and EBV copy number in the PCAWG cohort. Supplementary Figure S2. EBV copy number analysis for various cancer tissues and their paired normal tissues from the PCAWG cohort. Supplementary Figure S3. PD‐L1 gene expression and PD‐L1 protein expression in each EBV subtype. Supplementary Figure S4. Association between in situ hybridization targeting EBER (EBER‐ISH)‐positive cell counts and EBV copy number in our cohort. [file CAM4-12-1122-s001.pdf]

Supplementary Information

Association of tumors having Epstein-Barr virus in surrounding lymphocytes with poor prognosis

Norikazu Yogi, Genki Usui, Keisuke Matsusaka, Masaki Fukuyo, Ryoji Fujiki, Motoaki Seki, Shigetsugu Takano, Hiroyuki Abe, Teppei Morikawa, Tetsuo Ushiku, Masayuki Ohtsuka, Atsushi Kaneda

Supplementary Tables

Supplementary Table S1. Primers for quantitative PCR.

Supplementary Table S2. Primers for colony PCR and Sanger sequencing.

Supplementary Figures

Supplementary Fig. S1. Association between EBV transcripts and EBV copy number in the PCAWG cohort.

Supplementary Fig. S2. EBV copy number analysis for various cancer tissues and their paired normal tissues from the PCAWG cohort.

Supplementary Fig. S3. *PD-L1* gene expression and PD-L1 protein expression in each EBV subtype.

Supplementary Fig. S4. Association between *in situ* hybridization targeting EBER (EBER-ISH)-positive cell counts and EBV copy number in our cohort.

Supplementary Table S1. Primers for quantitative PCR.

	Primer type	Primer sequence	Annealing temperature (°C)	Amplicon size (bp)
EBNA1	Fwd	ACAAACCCTTGTACTGCGCTTG	61	77
	Rev	AAACTGGGCTTTCGCGTCATC		
LMP2A	Fwd	TCAGTTCTGGTGATGCTTGTGC	60	80
	Rev	ATGATGCCGCCACAAACAGTC		
CTRL	Fwd	AAGCTGGCACCACTACTTCAG	59	78
	Rev	TTTGGCCAACAGCAGATAGC		

CTRL, control region of host genome.

Supplementary Table S2. Primers for colony PCR and Sanger sequencing

	Primer sequence	Annealing temperature (°C)
SP6 promoter	GCCAAGCTATTTAGGTGACACTATAG	56
T7 promoter	GTGAATTGTAATACGACTCACTATAGGG	

Fig. S1

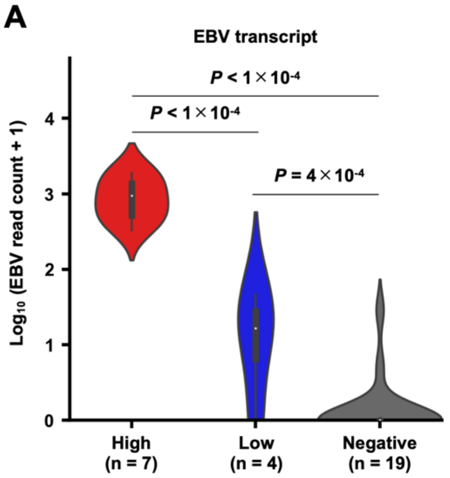

B

EBV copy number	EBV transcript (mean ± SD)
EBV-High (n = 7)	1015 ± 645
EBV-Low (n = 4)	20 ± 19
EBV-Negative (n = 19)	1 ± 6

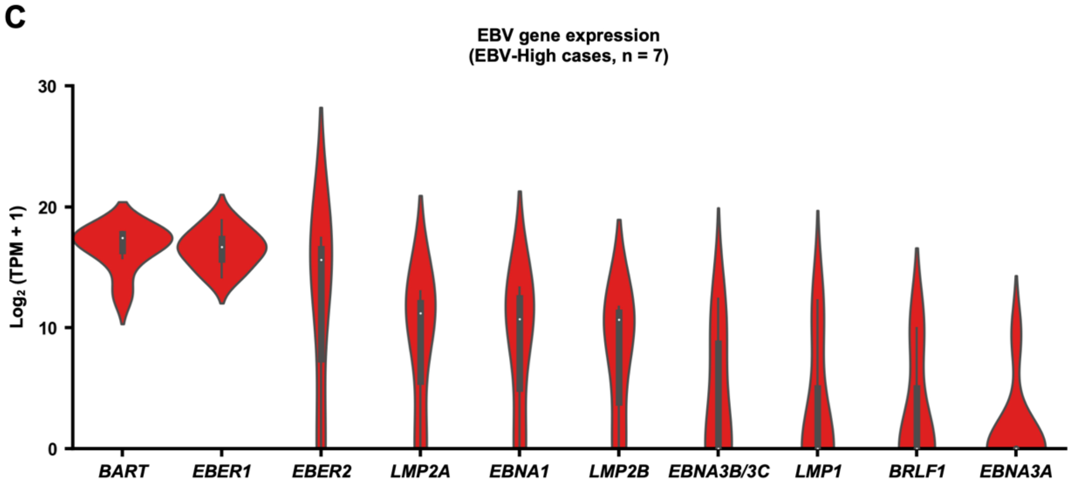

Supplementary Fig. S1. Association between EBV transcripts and EBV copy number in the PCAWG cohort.

(A, B) EBV transcript level among each EBV subtype in the PCAWG gastric cancer (GC) cohort (n = 30). The amount of EBV transcript in EBV-High cases was significantly higher than that in EBV-Low and EBV-Negative cases. The amount of EBV transcript in EBV-Low cases was significantly higher than that in EBV-Negative cases. **(C)** EBV gene expression level of EBV-High cases (n = 7) in the PCAWG GC cohort. EBV genes corresponding to latency type I were highly expressed in EBV-High cases.

Fig. S2

A

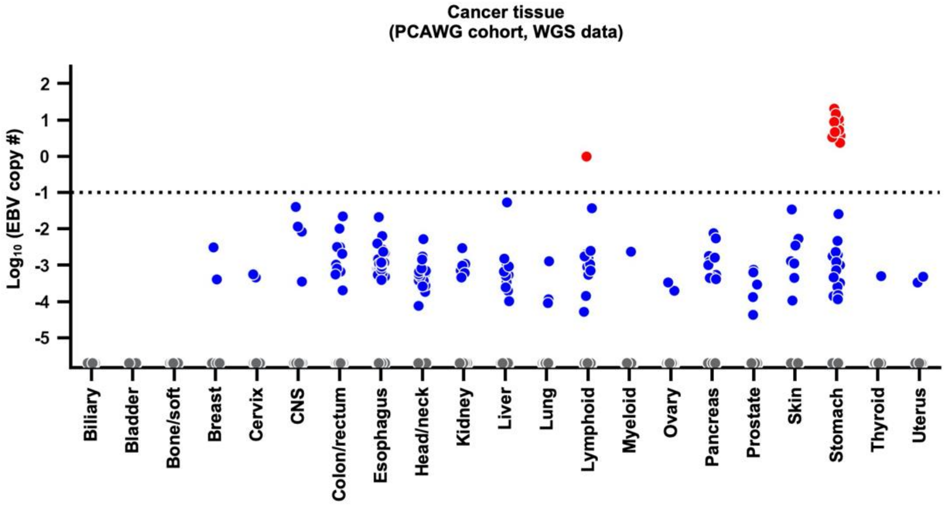

	Biliary	Bladder	Bone/soft	Breast	Cervix	CNS	Colon/rectum	Esophagus	Head/neck	Kidney	Liver	Lung	Lymphoid	Myeloid	Ovary	Pancreas	Prostate	Skin	Stomach	Thyroid	Uterus
● High	0	0	0	0	0	0	0	0	0	0	0	0	1	0	0	0	0	0	11	0	0
● Low	0	0	0	2	2	4	10	23	16	6	13	3	11	1	2	10	5	7	15	1	2
● Negative	12	23	98	212	18	290	50	74	41	183	317	83	190	65	111	316	254	100	49	47	49

B

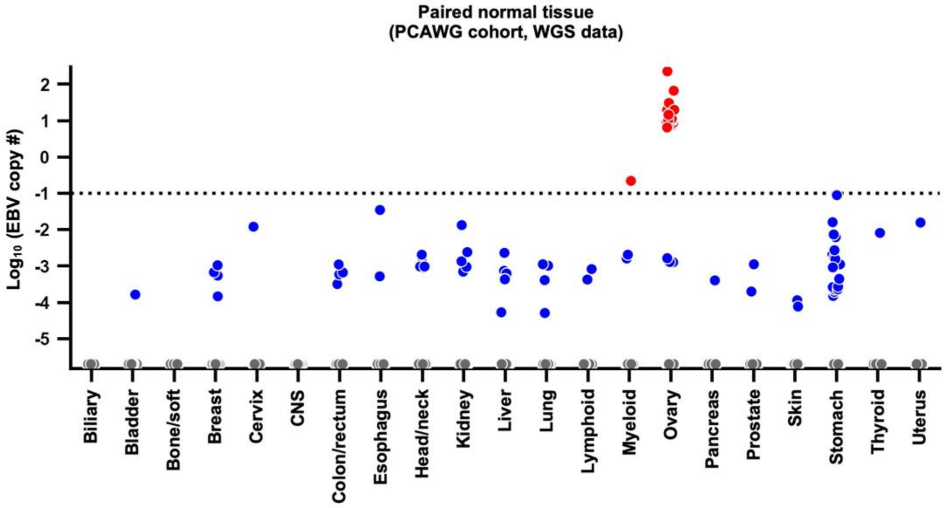

	Biliary	Bladder	Bone/soft	Breast	Cervix	CNS	Colon/rectum	Esophagus	Head/neck	Kidney	Liver	Lung	Lymphoid	Myeloid	Ovary	Pancreas	Prostate	Skin	Stomach	Thyroid	Uterus
● High	0	0	0	0	0	0	0	0	0	0	0	0	0	1	16	0	0	0	0	0	0
● Low	0	1	0	4	1	0	4	2	5	5	6	4	2	2	3	1	2	2	19	1	1
● Negative	12	22	97	210	19	284	56	96	52	183	315	82	200	35	94	322	208	105	56	47	50

Supplementary Fig. S2. EBV copy number analysis for various cancer tissues and their paired normal tissues from the PCAWG cohort.

(A) Distribution of EBV copy numbers in various cancer tissues (n = 2,727) using the whole-genome sequencing (WGS) data from the PCAWG cohort. The horizontal dotted line shows the cut-off value. *Red dots*, samples with high copy numbers of EBV. *Blue dots*, samples with low copy numbers of EBV. *Grey dots*, samples without EBV. **(B)** Distribution of EBV copy numbers in various normal tissues (n = 2,627) using the WGS data from the PCAWG cohort. EBV-High sample paired with myeloid leukemia was derived from saliva. EBV-High samples paired with ovarian cancer were derived from normal blood controls immortalized by EBV. The horizontal dotted line shows the cut-off value. *Red dots*, samples with high copy numbers of EBV. *Blue dots*, samples with low copy numbers of EBV. *Grey dots*, samples without EBV. *Bone/soft*, bone and soft tissue; *CNS*, central nervous system.

Fig. S3

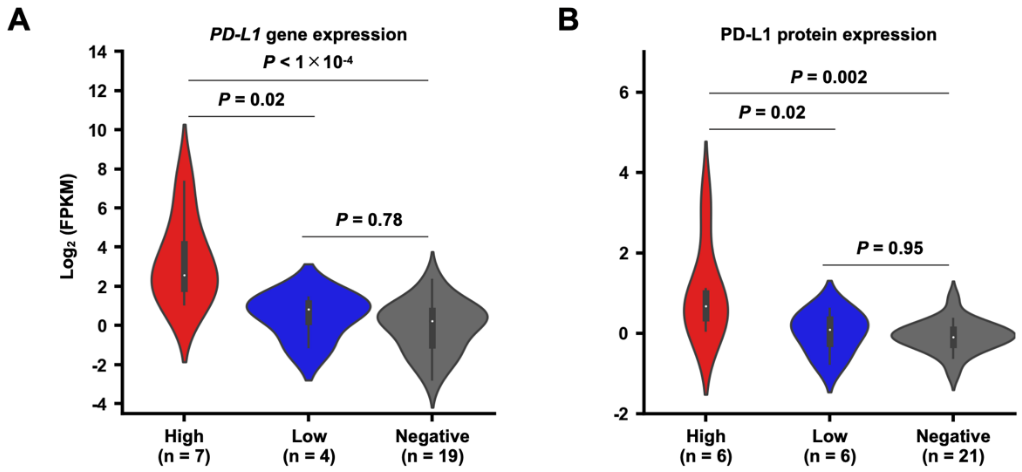

Supplementary Fig. S3. *PD-L1* gene expression and PD-L1 protein expression in each EBV subtype.

(A) *PD-L1* gene expression in each EBV subtype from PCAWG gastric cancer (GC) cohort (n = 30). EBV-High cases showed significantly higher *PD-L1* gene expression than EBV-Low cases. **(B)** PD-L1 protein expression in each EBV subtype from PCAWG GC cohort (n = 33). EBV-Low cases showed significantly higher PD-L1 protein expression than EBV-Low cases.

Fig. S4

A

EBV copy number — Stomach	EBER-ISH-positive cell count (mean ± SD)
High (n = 4)	2450 ± 1791
Low (n = 3)	10 ± 9
Negative (n = 85)	0 ± 0

B

EBV copy number — Pancreas	EBER-ISH-positive cell count (mean ± SD)
Low (n = 3)	6 ± 2
Negative (n = 102)	0 ± 0

C

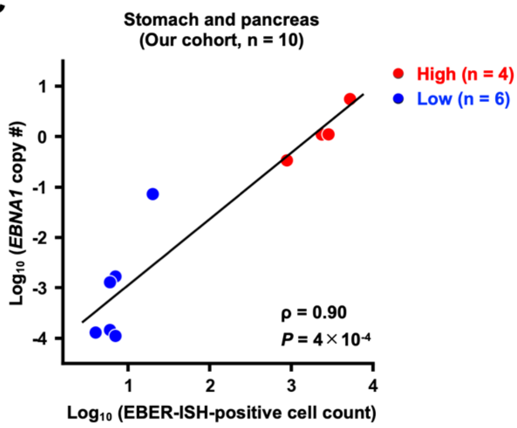

D

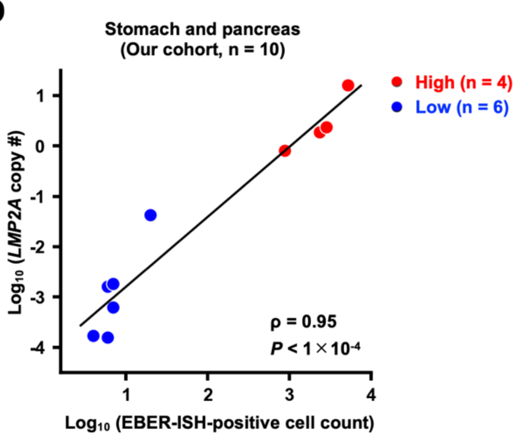

Supplementary Fig. S4. Association between *in situ* hybridization targeting EBER (EBER-ISH)-positive cell counts and EBV copy number in our cohort.

(A) EBER-ISH-positive cell counts in our gastric cancer (GC) cohort (n = 92). **(B)** EBER-ISH-positive cell counts in our pancreatic cancer (PC) cohort (n = 105). **(C)** Correlation between EBER-ISH-positive cell counts and *EBNA1* copy number in our GC and PC cohort. **(D)** Correlation between EBER-ISH-positive cell counts and *LMP2A* copy number in our GC and PC cohort.
